# Analysis of a large food chemical database: chemical space, diversity, and complexity

**DOI:** 10.12688/f1000research.15440.2

**Published:** 2018-08-10

**Authors:** J. Jesús Naveja, Mariel P. Rico-Hidalgo, José L. Medina-Franco

**Affiliations:** 1 PECEM, Faculty of Medicine, Universidad Nacional Autónoma de México, Mexico City, 04510, Mexico; 2 Department of Pharmacy, School of Chemistry, Universidad Nacional Autónoma de México, Mexico City, 04510, Mexico

**Keywords:** ChemMaps, chemical space, chemoinformatics, consensus diversity plots, diversity, FooDB, Foodinformatics, in silico

## Abstract

**Background:** Food chemicals are a cornerstone in the food industry. However, its chemical diversity has been explored on a limited basis, for instance, previous analysis of food-related databases were done up to 2,200 molecules. The goal of this work was to quantify the chemical diversity of chemical compounds stored in FooDB, a database with nearly 24,000 food chemicals.

**Methods:** The visual representation of the chemical space of FooDB was done with ChemMaps, a novel approach based on the concept of chemical satellites. The large food chemical database was profiled based on physicochemical properties, molecular complexity and scaffold content. The global diversity of FooDB was characterized using Consensus Diversity Plots.

**Results:** It was found that compounds in FooDB are very diverse in terms of properties and structure, with a large structural complexity. It was also found that one third of the food chemicals are acyclic molecules and ring-containing molecules are mostly monocyclic, with several scaffolds common to natural products in other databases.

**Conclusions:** To the best of our knowledge, this is the first analysis of the chemical diversity and complexity of FooDB. This study represents a step further to the emerging field of “Food Informatics”. Future study should compare directly the chemical structures of the molecules in FooDB with other compound databases, for instance, drug-like databases and natural products collections. An additional future direction of this work is to use the list of 3,228 polyphenolic compounds identified in this work to enhance the on-going polyphenol-protein interactome studies.

## Introduction

Despite the high relevance of food chemicals in many areas including nutrition, disease prevention, and broad impact in the food industry, the chemical space and diversity of food chemical databases ( Minkiewicz *et al*., 2016) has been quantified on a limited basis. Previous efforts include the analysis and comparison of about 2,200 Generally Recognized as Safe (GRAS) flavoring substances (discrete chemical entities only) with compound databases relevant in drug discovery and natural product research e.g., drugs approved for clinical use, compounds in the ZINC database, and natural products from different sources ( Burdock & Carabin, 2004; González-Medina *et al*., 2016; González-Medina *et al*., 2017; Martinez-Mayorga *et al*., 2013; Medina-Franco *et al*., 2012; Peña-Castillo *et al*., 2018). Other food-related chemical databases, comprising around 900 compounds, were analyzed by Ruddigkeit and J.-L. Reymond ( Ruddigkeit & Reymond, 2014). The limited quantitative analysis of food chemicals has been in part due to the scarce availability of food chemical databases in the public domain. A major exception, however, is FooDB a large database with more than 20,000 food chemicals ( The Metabolomics Innovation Centre, 2017). To date, it is the most informative public repository of food compounds.

As part of a continued effort to characterize the chemical contents and diversity of food chemicals ( González-Medina *et al*., 2016; Martinez-Mayorga & Medina-Franco, 2009; Medina-Franco *et al*., 2012), herein we report a quantitative analysis of the chemical space and chemical diversity of FooDB. Widely characterized compound databases such as GRAS, approved drugs and screening compounds used in drug discovery projects were employed as references. We used well-established and novel (but validated) chemoinformatic methods to analyze compound collections. Although most of these approaches are commonly used in drug discovery, this and previous works show they can be readily applied for food chemicals ( Peña-Castillo *et al*., 2018). Thereby this study represents a contribution to further advance the emerging field of Foodinformatics ( Martinez-Mayorga & Medina-Franco, 2014).

## Methods

### Databases and data curation

Four chemical databases were homogeneously curated and analyzed, namely: FooDB version 1.0 (accessed November, 2017) ( The Metabolomics Innovation Centre, 2017), drugs approved for clinical use available in DrugBank 5.0.2. ( Law *et al*., 2014), GRAS ( Burdock & Carabin, 2004), and a random subset of drug-like natural products from ZINC 12 ( Irwin & Shoichet, 2005), of a size comparable to FooDB. The GRAS and DrugBank sets used in this work also have been used as reference in other comparative studies ( Medina-Franco *et al*., 2012). The random set from ZINC was employed just as reference and other random sets from ZINC could be used. Compounds from all databases were washed and prepared using Wash MOE 2017 node in KNIME version 3.5.3 ( Berthold *et al*., 2008). Briefly, the washing protocol implemented in MOE included removing salts and neutralizing the charges in the molecules. The largest fragments were kept and duplicates in each dataset deleted. Table 1 summarizes the databases and sizes after data preprocessing.

**Table 1.** Compound databases analyzed in this work.

Database	Size ^a^
FooDB	23,883
GRAS	2,244
DrugBank	8,748
Natural products in ZINC (drug-like random subset)	24,000

^a^Number of compounds after data curation GRAS: Generally Recognized as Safe

### Chemical space visualization

The visual representation was generated with ChemMaps, a novel method for large chemical space visualizations ( Naveja & Medina-Franco, 2017). Briefly, ChemMaps is able to generate two- and three-dimensional representations of the chemical space based. It uses as input the pairwise chemical similarity computed using fingerprints data. This approach exploits the ‘chemical satellites’ concept ( Oprea & Gottfries, 2001), i.e., molecules whose similarity to the rest of the molecules in the database yield sufficient information for generating a visualization of the chemical space. Further details of ChemMaps are described elsewhere ( Naveja & Medina-Franco, 2017).

### Physicochemical properties

Six physicochemical properties (PCP) were calculated with RDKit KNIME nodes version 3.4, namely: SlogP (partition coefficient), TPSA (topological polar surface area), AMW (atomic mass weight), RB (rotatable bonds), HBD (hydrogen bond donors) and HBA (hydrogen bond acceptors). For the analysis reported in this short communication, these properties were selected based on their broadly extended use for cross-comparison of compound databases of biological relevance. However, additional properties can be calculated.

### Molecular complexity

Fraction of sp ^3^ carbons and number of stereocenters were computed for FooDB as measures of structural complexity. Despite the fact that there are several other measures, these two are straightforward to interpret, easy to calculate and are becoming standard to make cross comparisons among databases ( Méndez-Lucio & Medina-Franco, 2017). As described in the Results and Discussion section, the computed values for FooDB were compared to literature data already reported for the reference data sets.

### Scaffold content

The term “molecular scaffold” is employed to describe the core structure of a molecule ( Brown & Jacoby, 2006). Different approaches have been proposed to consistently obtain a molecule’s scaffold *in silico*. In this work, scaffolds were generated under the Bemis-Murcko definition using the RDKit nodes available in KNIME ( Bemis & Murcko, 1996). Bemis and Murcko define a scaffold as “the union of ring systems and linkers in a molecule”, i.e., all side chains of a molecule are removed.

### Global diversity

The so-called “global diversity” (or total diversity) of FooDB was assessed and compared to other reference collections using a consensus diversity plot ( González-Medina *et al*., 2016). As described recently, a consensus diversity plot simultaneously represents, in two-dimensions, four diversity criteria: structural (based on pairwise molecular fingerprint similarity values), scaffolds (using Murcko scaffolds computed as described in the Scaffold content section), physicochemical properties (based on the six properties described in Physicochemical properties section), and database size (the number of compounds) ( González-Medina *et al*., 2016). The structural diversity of each data set is represented on the X-axis and was defined as the median Tanimoto coefficient of MACCS keys fingerprints. The scaffold diversity of each database is represented on the Y-axis and was defined as the area under the corresponding scaffold recovery curve, a well-established metric to measure scaffold diversity ( Medina-Franco *et al*., 2009). The diversity based on PCP was defined as the Euclidean distance of six auto-scaled properties (SlogP, TPSA, AMW, RB, HBD, and HBA - *vide supra*) and is shown as the filling of the data points using a continuous color scale. The relative number of compounds in the data set is represented with a different size of the data points (smaller data sets are represented with smaller data points).

## Results and discussion

### Visual representation of the chemical space

**Figure 1.**
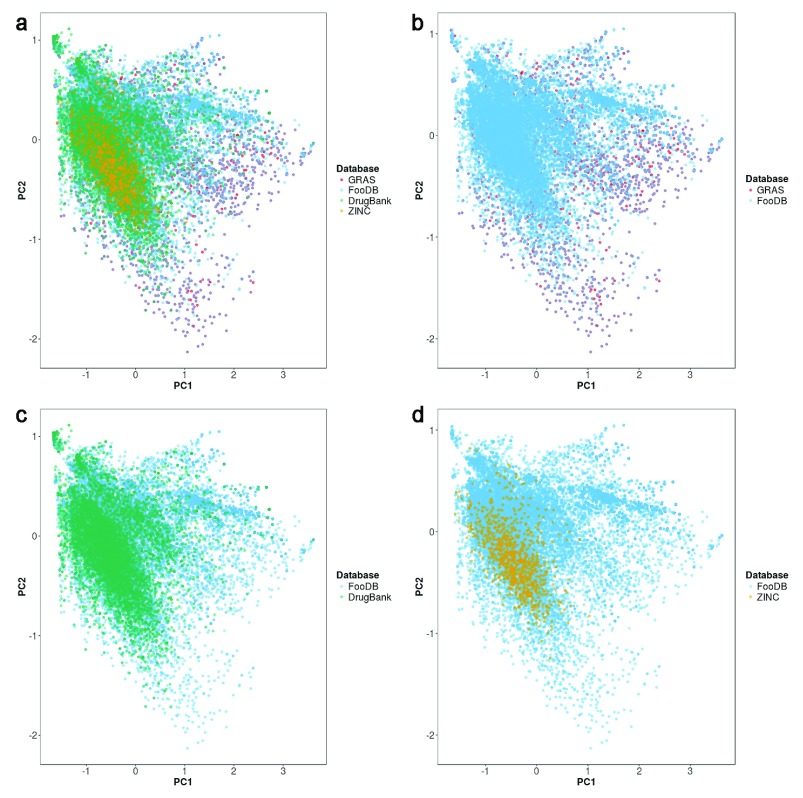
Representation of the chemical space of FooDB. The visual representation was generated with ChemMaps ( Naveja & Medina-Franco, 2017). **a**) Comparison of FooDB with three reference collections. Panels **b**– **d**) show comparisons of FooDB with individual data sets.

Chemical space of FooDB in comparison with the compounds of the three reference databases is visualized in Figure 1. The figure also shows the individual comparisons of FooDB with GRAS, DrugBank and natural products subset from ZINC, respectively. As shown in Figure 1a, the coverage of chemical space of FooDB is quite large as compared to other datasets. Most GRAS compounds lie within the chemical space framed by FooDB ( Figure 1b): indeed, 1,193 compounds (53% of GRAS) are structurally identical between the two databases. Hence, FooDB largely contains and upgrades structural information from GRAS. There is significant overlap with approved drugs ( Figure 1c) and natural products from ZINC with FooDB ( Figure 1d).

### Distribution of physicochemical properties

**Figure 2.**
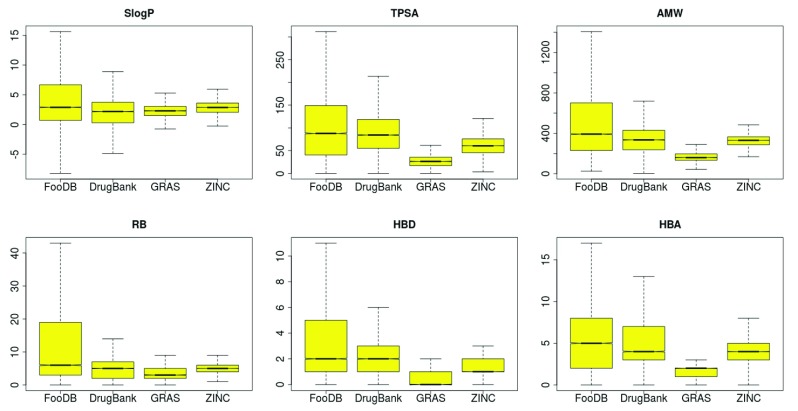
Distribution of physicochemical properties. Box plots of the distribution of six physicochemical properties of FooDB and reference data sets. SlogP (partition coefficient), TPSA (topological polar surface area), AMW (atomic mass weight), RB (rotatable bonds), HBD (hydrogen bond donors) and HBA (hydrogen bond acceptors).

Figure 2 shows the boxplots for the distribution of PCP in all the four databases. For better visualization, the outliers above or below the median +/- 1.5 interquartile range are omitted. As expected, due to the large structural diversity, distribution of PCP in FooDB is broad, in many cases overcoming even approved drugs. For most properties, except RB, several compounds in FooDB share the properties of drugs, and drug-like natural products in ZINC. The comparable physicochemical properties between compounds from FooDB and DrugBank encourages additional systematic investigations for bioactivity of food components. Of course, during this search one needs to consider that compounds with similar properties may have different activity profile. In turn, GRAS consists mostly of small-sized compounds. Table S1 ( Supplementary File 1) summarizes the statistics for FooDB and other reference collections.

### Molecular complexity

For FooDB, the fraction of sp ^3^ carbons (mean: 0.62; standard deviation: 0.28) and the number of stereocenters (mean: 4.7; standard deviation: 7.1) indicated a high structural complexity. For comparison, it has reported that the mean of the fraction of sp ^3^ carbons for approved drugs, compounds in the clinic and a general screening collections of organic compounds is 0.47, 0.41 and 0.32, respectively ( González-Medina *et al*., 2016; Lovering *et al*., 2009). Moreover, the reported mean of the fraction of sp3 carbons for natural products collections ranges between 0.41 and 0.58 (for natural products in ZINC and Traditional Chinese Medicine ( López-Vallejo *et al*., 2012). The complexity of compounds in FooDB is comparable to molecules in GRAS (mean: 0.63; standard deviation: 0.28) ( González-Medina *et al*., 2016).

### Scaffold content

**Figure 3.**
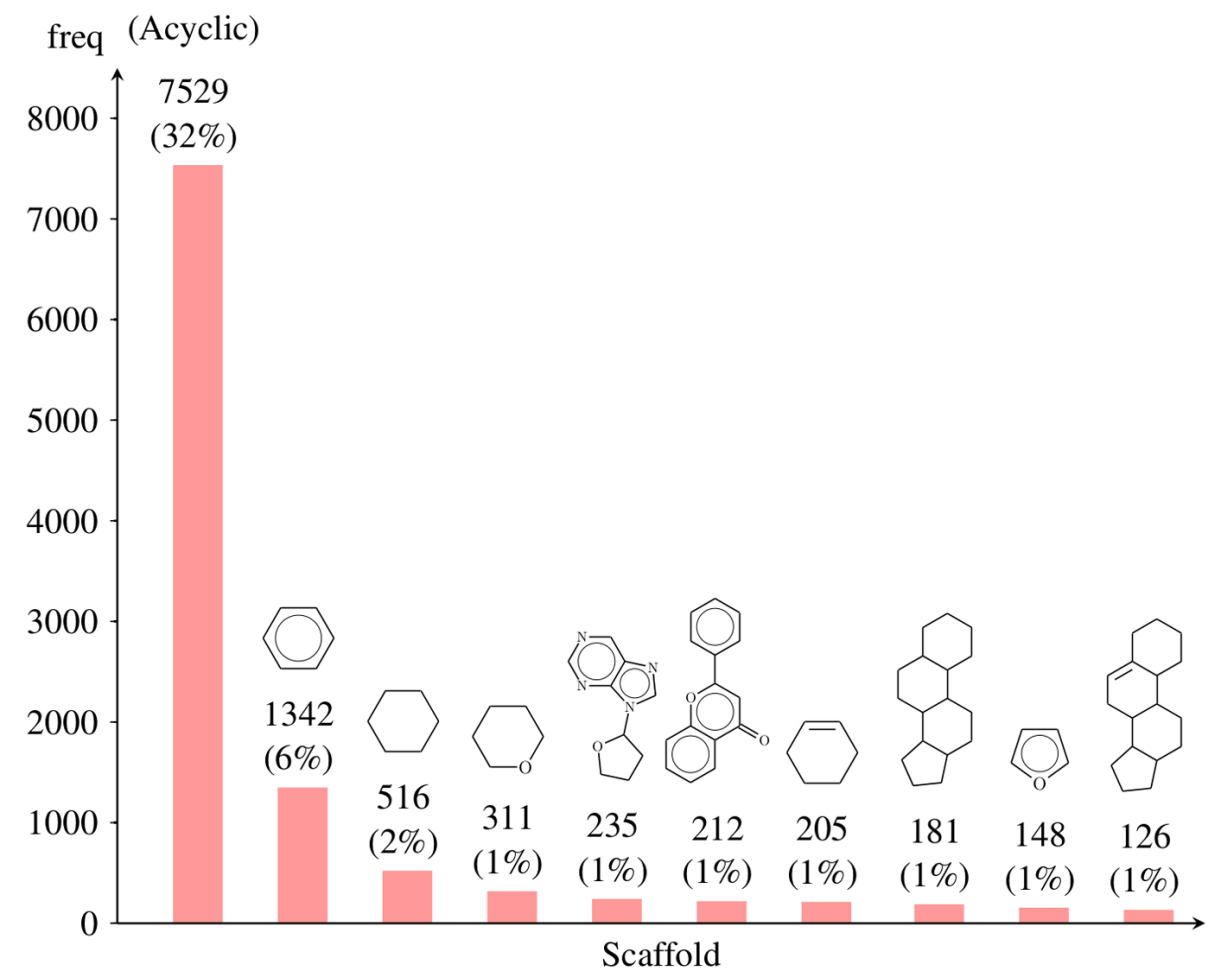
Frequency of the ten most common scaffolds in FooDB.

Figure 3 shows the frequency of the most common scaffolds in FooDB. Many compounds are acyclic (32%), followed by monocyclic compounds with a benzene (6%), cyclohexene (2%) and tetrahydropyran (1%) as a core structure. The benzene ring is the most common core scaffold in chemical databases used in drug discovery ( Bemis & Murcko, 1996; Singh *et al*., 2009; Yongye *et al*., 2012). Many of the most frequent scaffolds in FooDB are also common in other compound databases of natural products ( González-Medina *et al*., 2017). In a follow-up work, it will be interesting to explore the type of functional groups commonly present in the acyclic structures of FooDB.

Recently, Schneider *et al.* published an analysis on the selectivity of Bemis-Murcko scaffolds based on public bioactivity data available in ChEMBL ( Schneider & Schneider, 2017). 78 of the 585 scaffolds reported therein were present in FooDB. The list of the 78 matching scaffolds, along with the original statistics calculated by Schneider *et al*., is made available as Dataset 1 ( Naveja *et al*., 2018a). Of note, the three most frequent scaffolds in FooDB (benzene, cyclohexane and tetrahydropyran, with more than 300 compounds - Figure 3) are matching scaffolds. Interestingly, the mean *Information content* (I) value of all 585 Schneider’s scaffolds is 2.8 (sd= 0.6), while the subset of the 78 scaffolds also present in FooDB has a mean I value of only 2.1 (sd = 0.7). Lower I values point towards more promiscuous scaffolds ( Schneider & Schneider, 2017), an expected finding given the nature of the database. As example, Table S2 ( Supplementary File 1) shows and discusses briefly the statistics for the three most frequent matching scaffolds.

***Polyphenols.*** Since polyphenols are an important class of compounds in food chemistry ( Rasouli *et al*., 2017), we investigated and quantified the amount of polyphenols in FooDB. Polyphenols are well-known antioxidants, which may play a role in the prevention of several diseases including type 2 diabetes, cardiovascular diseases, and some types of cancer ( Neveu *et al*., 2010). In this line, it is known that oxidative/nitrosative stress has a pivotal role in pathophysiology of neurodegenerative disorders and other kinds of disease ( Ebrahimi & Schluesener, 2012). Polyphenols have been demonstrated to elicit several biological effects in *in vitro* and *ex vivo* tests ( Del Rio *et al*., 2010; Scalbert *et al*., 2005).

The molecular structure of polyphenols includes at least two phenolic groups, or one biphenol, and up to any additional number of OH substitutions in aryl rings. They may be classified by their structure in two major groups: flavonoids and non-flavonoids (phenolic acid derivatives) ( Del Rio *et al*., 2013). Some polyphenols, such as quercetin, are found in all plant products, whereas others are specific to particular foods. In many cases, food contain complex mixtures of polyphenols, which are often poorly characterized ( Manach *et al*., 2004).

Polyphenols are also a common chemical motif among natural products, and they are often associated to promiscuity ( Tang, 2016). In this work it was found that 3,228 (13.5%) compounds in FooDB are polyphenolic. The list of all 3,228 polyphenolic compounds is made available as Dataset 2 ( Naveja *et al*., 2018b). This set of polyphenols is larger than the 502 polyphenols from food indexed in Phenol-Explorer ( Neveu *et al*., 2010). For comparison, all the reference databases used in this work contained less polyphenols than FooDB. GRAS, ZINC and DrugBank contained 15 (0.6%), 24 (0.1%) and 325 (3.7%) polyphenols, respectively. The large list of polyphenols identified from FooDB is larger than the list of 1,395 polyphenols identified and used in the recent work of Lacroix *et al*. ( Lacroix *et al*., 2018) that was retrieved from Phenol-Explorer and the Dictionary of Natural Products. Indeed, the list of 3,228 polyphenolic compound made available in this work can be used to augment the already extensive polyphenol-protein interactome work of Lacroix *et al*. ( Lacroix *et al*., 2018).

### Global diversity

**Figure 4.**
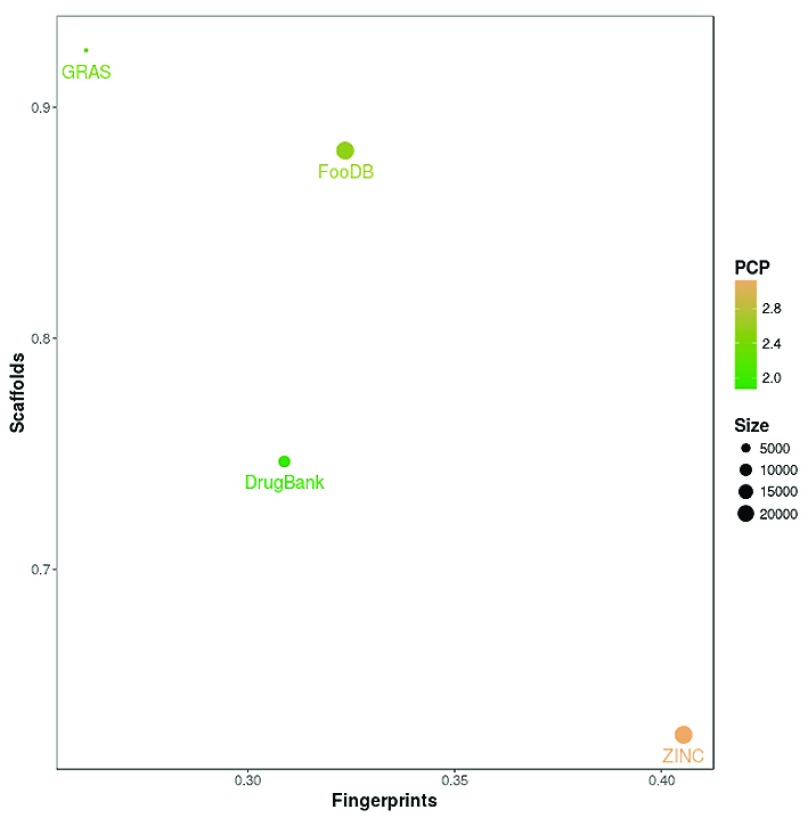
Consensus Diversity Plot of FooDB and reference data sets. The structural diversity of each data set is represented on the X-axis and was defined as the median Tanimoto coefficient of MACCS keys fingerprints. The scaffold diversity of each database is represented on the Y-axis and was defined as the area under the corresponding scaffold recovery curve. The diversity based on physicochemical properties (PCP) was defined as the Euclidean distance of six auto-scaled properties (SlogP, TPSA, AMW, RB, HBD, and HBA) and is shown as the filling of the data points using a continuous color scale. The relative number of compounds is represented with a different size of the data points (smaller data sets are represented with smaller data points).

Since the diversity of compound data sets depend on the molecular representation ( Sheridan & Kearsley, 2002), a global assessment of the diversity of FooDB was analyzed using different criteria: molecular fingerprints, scaffolds, physicochemical properties and number of compounds. The four criteria were analyzed in an integrated manner through a Consensus Diversity Plot generated as described in the Global diversity section of the Methods. The Consensus Diversity Plot in Figure 4 shows that FooDB has about average diversity both by fingerprints and relatively low diversity by scaffolds. Although PCP (represented with the color of the data points) are extremely diverse, structural motifs seem to reappear with slight variations. Figure 4 shows the overall large fingerprint and scaffold diversity of approved drugs (e.g., data points towards the lower left region of the plot). Similarly, the relative global diversity of GRAS i.e., high fingerprint diversity but low scaffold diversity (e.g., upper left region of the plot), is consistent with previous comparisons of these compounds with other reference data sets ( González-Medina *et al*., 2016; Medina-Franco *et al*., 2012).

Schneidermatch.sdf. This file contains the list of the 78 matching scaffolds in SDF format, along with the original statistics calculated by Schneider *et al.* No special software is required to open the SDF files. Any commercial or free software capable of reading SDF files will open the data sets supplied Click here for additional data file. Copyright: © 2018 Naveja JJ et al. 2018 Data associated with the article are available under the terms of the Creative Commons Zero "No rights reserved" data waiver (CC0 1.0 Public domain dedication).

FooDBpolyphenols.sdf. This file contains 3,228 polyphenolic compounds available in FooDB, in SDF format No special software is required to open the SDF files. Any commercial or free software capable of reading SDF files will open the data sets supplied Click here for additional data file. Copyright: © 2018 Naveja JJ et al. 2018 Data associated with the article are available under the terms of the Creative Commons Zero "No rights reserved" data waiver (CC0 1.0 Public domain dedication).

## Conclusions

FooDB is a novel, large and diverse library containing information of more than 23,000 compounds found in food. To date, it is the most informative public resource of food compounds. Visual representation of the chemical space revealed that FooDB largely contains and upgrades structural information from GRAS. Indeed, most of GRAS is contained in FooDB. Compounds in FooDB have a large diversity of physicochemical properties. The distributions of most physicochemical properties of FooDB compounds overlap with those of approved drugs and natural products in ZINC. GRAS mostly contains small-sized compounds. The global diversity indicates that FooDB has a large structural diversity as measured by molecular fingerprints, though it has relatively low scaffold diversity. One third of the compounds in FooDB are acyclic. The most frequent cyclic scaffolds are monocyclic. Of note, polyphenols represent a large fraction of FooDB. The list of 3,228 polyphenolic compounds identified in this work to enhance the on-going polyphenol-protein interactome studies. Analysis of the chemical complexity revealed that compounds in FooDB are more complex than approved drugs and natural products and have complexity comparable to GRAS compounds. A next step of this work is to compare the chemical space of FooDB with that of natural products from different sources, e.g., plants, terrestrial, cyanobacteria. A second suggested future study is to perform the virtual screening of FooDB across a range of targets, for instance, the increasingly important epigenetic targets ( Naveja & Medina-Franco, 2018). Virtual screening can be done using multiple methods, for instance, using similarity searching. In this case one needs to consider, however, the potential presence of activity cliffs i.e., compounds with similar structure but different activity ( Stumpfe *et al*., 2014). The goal of such study would be to identify systematically dietary components that may be participating in epigenetic regulatory processes ( Martinez-Mayorga *et al*., 2013). These efforts are ongoing in our group and will be reported in due course. Other perspective of this work is integrating the knowledge of FooDB with other large databases with the aim of identifying food-disease associations and food-drug interactions such as the works previously published by Jensen *et al*. ( Jensen *et al*., 2014; Jensen *et al*., 2015).

## Data availability

The data referenced by this article are under copyright with the following copyright statement: Copyright: © 2018 Naveja JJ et al.

Data associated with the article are available under the terms of the Creative Commons Zero "No rights reserved" data waiver (CC0 1.0 Public domain dedication). 

**Dataset 1:** (Schneidermatch.sdf). **This file contains the list of the 78 matching scaffolds in SDF format**, along with the original statistics calculated by Schneider *et al*. No special software is required to open the SDF files. Any commercial or free software capable of reading SDF files will open the data sets supplied. 10.5256/f1000research.15440.d209071 ( Naveja, *et al.*, 2018a)

**Dataset 2:** (FooDBpolyphenols.sdf). **This file contains 3,228 polyphenolic compounds available in FooDB, in SDF format**. No special software is required to open the SDF files. Any commercial or free software capable of reading SDF files will open the data sets supplied. 10.5256/f1000research.15440.d209072 ( Naveja *et al*., 2018b)

## Supplementary material

**Supplementary File 1: File with supporting tables**. Table S1: Summary statistics of the distribution of six PCP of FooDB and other reference collections. Table S2: Selected scaffold statistics as reported by ( Schneider & Schneider, 2017).
