# Supplementary material for: Analysis of a large food chemical database: chemical space, diversity, and complexity
Source: F1000Res. 2018 Aug 10;7:Chem Inf Sci-993. Originally published 2018 Jul 3. [Version 2] doi: 10.12688/f1000research.15440.2 (PMC6081979; doi:10.12688/f1000research.15440.2)
Supplement: Supplementary file 3 [file f1000research-7-17367-s0002.tgz › 8f3fd486-d217-4dcf-9180-8e512d165bae.docx]

**Supplementary data**

**Analysis of a large food chemical database: Chemical space, diversity, and complexity**

J. Jesús Naveja,^1,2^ Mariel P. Rico-Hidalgo,^1^ José L. Medina-Franco*^1,2^

*^1^Department of Pharmacy, School of Chemistry, Universidad Nacional Autónoma de México, Mexico City, 04510, Mexico*

*^2^PECEM, Faculty of Medicine, Universidad Nacional Autónoma de México, Mexico City, 04510, Mexico*

**Table S1.** Summary statistics of the distribution of six PCP of FooDB and other reference collections.

| Property | FooDB | GRAS | ZINC | DrugBank |
| --- | --- | --- | --- | --- |
| SlogP | mean: 4  sd: 5.3  median: 2.9 | mean: 2.3  sd: 1.3  median: 2.3 | mean: 2.8  sd: 1.2  median: 2.9 | mean: 1.9  sd: 3  median: 2.2 |
| TPSA | mean: 124.7  sd: 129.4  median: 87.7 | mean: 29  sd: 24.3  median: 26.3 | mean: 61  sd: 22  median: 60.6 | mean: 102.9  sd: 108.3  median: 84.3 |
| AMW | mean: 490  sd: 344.9  median: 392.6 | mean: 169.9  sd: 61.9  median: 160.2 | mean: 328.9  sd: 61.3  median: 331.4 | mean: 365.7  sd: 263.4  median: 335.3 |
| RB | mean: 13.6  sd: 16  median: 6 | mean: 3.9  sd: 3.1  median: 3 | mean: 4.6  sd: 1.5  median: 5 | mean: 5.8  sd: 7.6  median: 5 |
| HBD | mean: 3.5  sd: 4.7  median: 2 | mean: 0.9  sd: 3.9  median: 3 | mean: 1.1  sd: 0.8  median: 1 | mean: 2.8  sd: 3.6  median: 2 |
| HBA | mean: 7.2  sd: 5  median: 7.2 | mean: 1.4  sd: 0.4  median: 0 | mean: 4.1  sd: 1.5  median: 4 | mean: 5.4  sd: 5.5  median: 4 |

SlogP: calculated partition coefficient; TPSA: topological polar surface area; AMW: atomic mass weight; RB: rotatable bonds; HBD: hydrogen bond donors; HBA: hydrogen bond acceptors.

**Table S2.** Selected scaffold statistics as reported by [(Schneider & Schneider, 2017)](http://f1000.com/work/citation?ids=3883310&pre=&suf=&sa=0).

| Structure | Compounds in ChEMBL 22 | Shannon entropy | Information | Kullback– Leibler divergence | Mean neural network score |
| --- | --- | --- | --- | --- | --- |
| 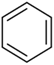 | 11,048 | 1.96 | 1.36 | 0.68 | 0.23 |
| 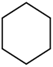 | 277 | 1.54 | 1.78 | 1.17 | 0.05 |
| 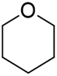 | 119 | 0.61 | 2.71 | 2.08 | 0 |

Briefly, to study the promiscuity or selectivity of chemical scaffolds, [(Schneider & Schneider, 2017)](http://f1000.com/work/citation?ids=3883310&pre=&suf=&sa=0)) compared the distribution of each scaffold activity annotations in different targets families (GPCR, enzyme, kinase, proteinase, nuclear receptor, catalytic receptor, ion channel, transporter, protein and undefined). Shannon entropy was calculated as an indicator of the variability (a higher value means more promiscuity). Information was the difference of the maximum possible entropy and the calculated Shannon entropy (a higher value means lower Shannon entropy, i.e., more selectivity). Kullback-Leibler divergence is another entropy measurement that considers the a priori unequal target distribution in the whole database (similar to Shannon entropy, a higher value corresponds to more promiscuity in general). Finally, a neural network trained to predict the promiscuity of the ligands was used, which provided a score proportional to the expected promiscuity of the ligand. It was shown that this score is linearly independent to entropy measurements.
