# Supplementary material for: Analysis of a large food chemical database: chemical space, diversity, and complexity
Source: F1000Res. 2018 Aug 10;7:Chem Inf Sci-993. Originally published 2018 Jul 3. [Version 2] doi: 10.12688/f1000research.15440.2 (PMC6081979; doi:10.12688/f1000research.15440.2)
Supplement: Peer review history

# Peer review: Referee response for version 2

**DOI:** 10.5256/f1000research.17367.r37062

**Published:** 2018-08-14

**Authors:** Piotr Minkiewicz

**Affiliations:** 1 Department of Food Biochemistry, Faculty of Food Science, University of Warmia and Mazury in Olsztyn, Olsztyn-Kortowo, Poland

I fully approve the recent version of the artcle. I can recommend it as very valuable for readers representing the areas of food science and pharmaceutical science.

I have read this submission. I believe that I have an appropriate level of expertise to confirm that it is of an acceptable scientific standard.

# Peer review: Referee response for version 1

**DOI:** 10.5256/f1000research.16825.r36226

**Published:** 2018-08-07

**Authors:** Rachelle J. Bienstock

**Affiliations:** 1 RJB Computational Modeling LLC, Chapel Hill, NC, USA

The paper on chemical diversity of FooDB compared to several other databases, including GRAS and DrugBank and drug-like natural products fromZINC12, by Naveja, Rico-Hidalgo, and Medina-Franco was an interesting, informative and nicely presented analysis.  The figures and graphical presentation of ChemMaps results in particular is very clear.  One thing which I think would be interesting for a further study and analysis, (since epigenetics and some other diseases and health implications are mentioned in regards to polyphenols) is an analysis regarding vitamins and other compounds and dietary supplements which have had specific health claims made.  ChemMaps analysis of these compounds according to properties in these databases and correlation with biological pathways would be interesting for future work.

I have read this submission. I believe that I have an appropriate level of expertise to confirm that it is of an acceptable scientific standard.

# Response

**Published:** 2018-08-07

**Authors:** José L. Medina-Franco

**Affiliations:** Universidad Nacional Autónoma de México, Mexico

We are grateful for the positive comments and thank the reviewer for the excellent suggestions to expand this work in future studies.

# Peer review: Referee response for version 1

**DOI:** 10.5256/f1000research.16825.r36288

**Published:** 2018-07-30

**Authors:** Khushbu Shah

**Affiliations:** 1 Division of Medicinal Chemistry, Graduate School of Pharmaceutical Sciences, Duquesne University, Pittsburgh, PA, USA; 2 Kramer Levin Naftalis Frankel LLP, New York, NY, USA

This manuscript purports to analyze and disclose the chemical diversity of the FooDB database. It is an interesting study with a logical flow based on appropriate methods.

 There a few *optional suggestions* that the authors could adapt in the manuscript:

- It would be advisable for the authors to add the rationale behind selecting the three versions – GRAS, DrugBank and ZINC for data curation.
- Since acyclic compounds represented the most common scaffold in FooDB, the authors could expand upon the types of functional groups commonly observed in these acyclic compounds in FooDB.
- Further, the authors point out that there are more polyphenols in FooDB vs. Phenol-explorer. The authors could include the dataset from Phenol-explorer in a consensus diversity plot (like Figure 4) to clearly represent their reults.  

I have read this submission. I believe that I have an appropriate level of expertise to confirm that it is of an acceptable scientific standard.

# Response

**Published:** 2018-08-07

**Authors:** José L. Medina-Franco

**Affiliations:** Universidad Nacional Autónoma de México, Mexico

We really appreciate the reviewer´s feedback and value the optional suggestions. In the revised manuscript we added the rationale for selecting the ´specific version of the three data sets. We also included a comment that a systematic analysis of the functional groups present in the acyclic structures is highly relevant. This excellent suggestion, as well as the comparison of the polyphenols in FooDB with those in Phenol-explorer, will be reported in a follow-up study.

# Peer review: Referee response for version 1

**DOI:** 10.5256/f1000research.16825.r35684

**Published:** 2018-07-11

**Authors:** Piotr Minkiewicz

**Affiliations:** 1 Department of Food Biochemistry, Faculty of Food Science, University of Warmia and Mazury in Olsztyn, Olsztyn-Kortowo, Poland

I have no critical remarks concerning methods, correctness of work. Discussion is also appropriate from the point of view of scientists working in the areas of cheminformatics and/or pharmacology.

 I would like to ask some questions concerning relevance of the article for food science.

 The analysis performed reveals similarity in structural and physico-chemical features between compounds from FooDB and DrugBank. Does it mean that more detailed studies may reveal similar biological activity (i.e. interactions with the same target) of drugs and bioactive food components.

 Are Authors’ results consistent with these published in the following articles concerning similarity of effects of drugs and food components?

 Jensen K. et al. PLoS Comput Biol, 10, (2014) ^1^

 Jensen K. et al. PLoS Comput Biol, 11, (2015) ^2^

 Proteins interacting with polyphenols and described in the following article: Lacroix S. et al. Sci Rep, 8, (2018) ^3^ are also annotated in DrugBank as drug targets. Is the above finding consistent with the Authors’ conclusions?

 I would like to ask Authors to add few sentences concerning limitations of the proposed methodology  (for instance limitations occurring due to presence of activity cliffs).

I have read this submission. I believe that I have an appropriate level of expertise to confirm that it is of an acceptable scientific standard, however I have significant reservations, as outlined above.

## References

[1] JensenKPanagiotouGKouskoumvekakiI: Integrated text mining and chemoinformatics analysis associates diet to health benefit at molecular level. *PLoS Comput Biol*.2014;10(1) : 10.1371/journal.pcbi.1003432 e1003432 10.1371/journal.pcbi.1003432 24453957PMC3894162

[2] JensenKNiYPanagiotouGKouskoumvekakiI: Developing a molecular roadmap of drug-food interactions. *PLoS Comput Biol*.2015;11(2) : 10.1371/journal.pcbi.1004048 e1004048 10.1371/journal.pcbi.1004048 25668218PMC4323218

[3] LacroixSKlicic BadouxJScott-BoyerMPParoloSMatoneAPriamiCMorineMJKaputJMocoS: A computationally driven analysis of the polyphenol-protein interactome. *Sci Rep*.2018;8(1) : 10.1038/s41598-018-20625-5 2232 10.1038/s41598-018-20625-5 29396566PMC5797150

# Response

**Published:** 2018-07-16

**Authors:** José L. Medina-Franco

**Affiliations:** Universidad Nacional Autónoma de México, Mexico

Thank the reviewer for critically reading our manuscript and the valuable feedback. Hereunder we provide a point-by-point response to each comment.

**Comment: **"I have no critical remarks concerning methods, correctness of work. Discussion is also appropriate from the point of view of scientists working in the areas of cheminformatics and/or pharmacology.

  I would like to ask some questions concerning relevance of the article for food science.

  The analysis performed reveals similarity in structural and physico-chemical features between compounds from FooDB and DrugBank. Does it mean that more detailed studies may reveal similar biological activity (i.e. interactions with the same target) of drugs and bioactive food components."

**Response**: We agree with the valuable input. Indeed, as the reviewers points out, similar physico-chemical properties between compounds from FoodDB and DrugBank encourages additional systematics investigations for bioactivity of food components. In the revised version of the manuscript, that is under editing and will be uploaded in due course, we will expand the discussion of the manuscript elaborating more on the significance of the work.

**Comment: ** "Are Authors’ results consistent with these published in the following articles concerning similarity of effects of drugs and food components?

  Jensen K. et al. PLoS Comput Biol, 10, (2014)1

  Jensen K. et al. PLoS Comput Biol, 11, (2015)2'

**Response**: We are grateful to the reviewer for pointing out the two papers of Jensen K. et al. As stated in the manuscript, the goal of this study was to characterize the chemical content, diversity and complexity of the chemical structures of a large and public database of food chemicals. The studies of Jensen et al. are focused on finding food-disease associations and food-drug interactions. Following the reviewers advice, we addressed this comment in the revised manuscript stating that as a Perspective of our current work, the FooDB can be used to further augment the current knowledge of food-disease associations and food-drug interactions. The two suggested references are being added to the revised manuscript.

**Comment: **"Proteins interacting with polyphenols and described in the following article: Lacroix S. et al. Sci Rep, 8, (2018)3 are also annotated in DrugBank as drug targets. Is the above finding consistent with the Authors’ conclusions?"

**Response:** Thank the reviewer for bring to our attention the work of Lacroix S. et al. Our results are consistent with this study. In particular, the number of polyphenol compounds found in the FooDB is larger than the amount of compounds found in the Phenol-Explorer database. This point is being addressed in section “ *3.4.1. Polyphenols” *of the revised manuscript. In the revised manuscript we added the suggested reference. In addition, in the Conclusions section, we are also stating that the set of polyhpenols from FooDB identified in this work can further enrich the on-going efforts of the polyphenol-protein interactome studies such as the one published by Lacroix S. et al.

**Comment: **"I would like to ask Authors to add few sentences concerning limitations of the proposed methodology (for instance limitations occurring due to presence of activity cliffs)."

**Response**: Following the reviewers’ advice, we added a discussion of the limitations of the methodology addressing the caution that needs to be taken while dealing with activity cliffs. Relevant references to activity cliffs are being added.
